# Employment of single-diode model to elucidate the variations in photovoltaic parameters under different electrical and thermal conditions

**DOI:** 10.1371/journal.pone.0182925

**Published:** 2017-08-09

**Authors:** Fahmi F. Muhammad, Mohd Y. Yahya, Shilan S. Hameed, Fakhra Aziz, Khaulah Sulaiman, Mariwan A. Rasheed, Zubair Ahmad

**Affiliations:** 1 Soft Materials and Devices Laboratory, Department of Physics, Faculty of Science & Health, Koya University, Koya, Kurdistan Region, Iraq; 2 Centre for Composites, Institute for Vehicle Systems & Engineering, Faculty of Mechanical Engineering, Universiti Teknologi Malaysia, Johor Bahru, Malaysia; 3 Department of Computer Science, Faculty of Computing, Universiti Teknologi Malaysia, Johor Bahru, Malaysia; 4 Department of Software & Informatics, College of Engineering, University of Salahaddin, Erbil, Kurdistan Region, Iraq; 5 Department of Electronics, Faculty of Physical and Numerical Sciences, University of Peshawar, Peshawar, Pakistan; 6 Low Dimensional Materials Research Centre, Department of Physics, Faculty of Science, University of Malaya, Kuala Lumpur, Malaysia; 7 Development Centre for Research and Training (DCRT), University of Human Development, Sulaimani, Kurdistan Region, Iraq; 8 Department of Electrical Engineering, College of Engineering, Qatar University, Doha, Qatar; Chongqing University, CHINA

## Abstract

In this research work, numerical simulations are performed to correlate the photovoltaic parameters with various internal and external factors influencing the performance of solar cells. Single-diode modeling approach is utilized for this purpose and theoretical investigations are compared with the reported experimental evidences for organic and inorganic solar cells at various electrical and thermal conditions. Electrical parameters include parasitic resistances (*R*_*s*_ and *R*_*p*_) and ideality factor (*n*), while thermal parameters can be defined by the cells temperature (*T*). A comprehensive analysis concerning broad spectral variations in the short circuit current (*I*_*sc*_), open circuit voltage (*V*_*oc*_), fill factor (*FF*) and efficiency (*η*) is presented and discussed. It was generally concluded that there exists a good agreement between the simulated results and experimental findings. Nevertheless, the controversial consequence of temperature impact on the performance of organic solar cells necessitates the development of a complementary model which is capable of well simulating the temperature impact on these devices performance.

## Introduction

The most promising way to tackle the limiting supply of today’s main energy sources and their detrimental impact on the environment is to harness solar energy. It is imperative to evolve complement strategies into the process of solar energy conversion and its storage management. Supercapacitors or ultracapacitors [1, 2] are considered to be feasible reservoirs for the storage of solar electricity power and advanced power-source integration [3, 4]. Additionally, the utilization of solar energy has become instrumental in the state-of-art technologies, targeting the reduction of carbon dioxide emissions and cost-effectiveness. For instance, Hu *et al*. [5] explored the role of renewable energy and powertrain optimization in minimizing daily carbon emissions of plug-in hybrid electric vehicles (PHEVs). Very recently, the integration of photovoltaic arrays with battery energy storage of PHEV for smart home energy management was also elaborated [6]. In these contexts, the exploitation of photovoltaic (PV) technology to convert sunlight energy into electricity through solar panels is increasingly demanded by the public, industries and space program sectors [7–9]. This is mainly due to easy installation and low maintenance cost of solar panels compared with those of other electricity sources [10]. Solar panels are made from solar cells connected in series and parallel schemes in order to provide the desired current and voltage. It is known that the performance of solar cells can be affected by the change in temperature, sunlight intensity and aging [11, 12]. Therefore, it is crucial to have a model capable of simulating the real behaviour of solar panels, through which a comprehensive investigation on the devices parameters can be realized. Researchers have widely examined the single-diode and double-diode model to simulate solar cells characteristics and to determine the devices parameters [13, 14]. Comparatively, the single-diode model, which is also known as five parameters model, demonstrates reasonable accuracy and simplicity in the parameters estimation of various solar cell technologies [15]. This model comprises of an ideal diode connected in parallel with a constant current source and a shunt resistance bypassed to the external load through a series resistance. The implication of single-diode model allows us to perform sensitivity analysis on the solar cell parameters, thereby taking valuable strategies towards the improvement of modelling capabilities [16]. Simulations can be interestingly used to analyse solar cells and to predict various internal and external effects due to device changes or ambient conditions. Hence, they may promote device optimization and provide potential information regarding viable improvements.

Solar cell parameters including series resistance (*R*_*s*_), parallel resistance (*R*_*p*_) and ideality factor (*n*) can be readily extracted from the single-diode model [17]. These parameters describe the internal properties of the devices raising during the process of fabrication. In practical considerations, low *R*_*s*_ and high *R*_*p*_ values are favoured to achieve enhanced solar cell performance. One of the main challenges in front of researchers and users of solar panels is the difficulty of estimating the operational performance of these devices at modified internal and/or external parameters due to the impact of fabrication process, aging and ambient temperature. Therefore, it is of great importance to investigate and analyse the correlation between photovoltaic parameters, which are key performance measure of solar panels, and aforementioned internal/external parameters (electrical and thermal). Furthermore, results of such investigations can be highly beneficial for enhanced prediction strategy and model’s building in the optimization of energy management and power smoothing of solar PV systems [18, 19], while optimized PV plant is complicated by oscillating PV output due to uncertain environmental conditions, internal resistance variations and aging. The concepts might be further applicable when maximum power point tracking (MPPT) techniques are considered for delivering optimum power to the loads [20]. To the best of our knowledge, little attention has been paid to perform a systematic investigation on the variation of photovoltaic parameters due to the impact of broad modifications in the coefficients of solar cells having various efficiencies. Hence, the current work is intended to report on the employment of single-diode model to elucidate the variations in photovoltaic parameters under the influence of internal and external factors such as parasitic resistances, ideality factor and temperature. The main contribution of this work is to reveal and understand the important correlations between solar cell parameters and their photovoltaic performance along with temperature impact on the devices having various efficiencies, by which effective improvement approaches can be made upon the production of these devices.

## Materials and methods

**Fig 1:**
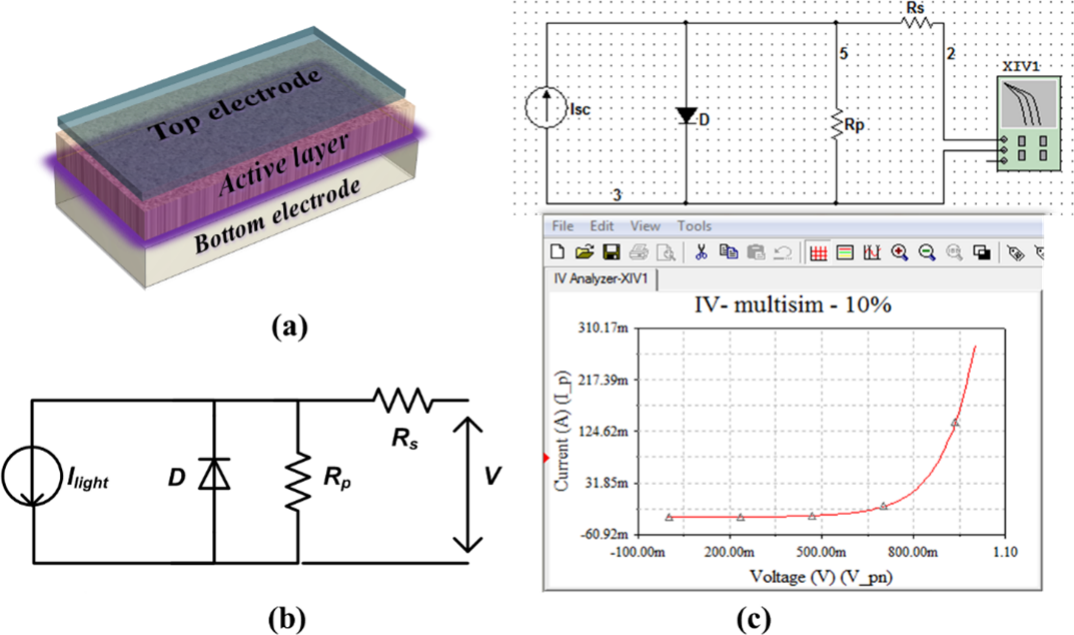
A prototype architecture of solar cells (a), the equivalent circuit used to model the *I-V* characteristics of the devices (b), and workbench view of the simulated equivalent circuit by using Multisim Power Pro.10.

A prototype structure of solar cells is shown in Fig 1(A), in which the active layer is made of a p-n bulk heterojunction responsible for absorbing sunlight energy and producing free electrons and holes. The bottom electrode is responsible to collect free holes, while the top electrode receives free electrons. Fig 1(B) represents the single-diode model which is capable of well modelling the current-voltage (*I-V*) characteristics of solar cells, while Fig 1(C) shows the circuit simulator layout designed by using Multisim Power Pro. 10 electronic workbench software. The light activated current source (*I*_*light*_) depicts the amount of current generated in the cell when it is exposed to sunlight energy. The application of a load across the right side of the circuit gives rise to a voltage induction, which ultimately acts upon reducing the total amount of current passing through it in a reverse biased direction. Hence, the instantaneous average current in the load defines the characteristic current of the solar cell. From the electrical circuit, one can easily estimate the net current as follows:

$$I=I_{s}\left[\exp\left(\frac{V-IR_{s}}{nK_{B}T/q}\right)-1\right]+\frac{V-IR_{s}}{R_{p}}-I_{light}$$ (1)

Where, *I*_*s*_ is the saturation current of the diode under dark, *K*_*B*_ is the Boltzmann’s constant, *T* is the temperature in Kelvin, *q* is electron unit charge, *R*_*s*_ and *R*_*p*_ are the series and parallel resistances of the device, respectively. The value of *n* in the equation defines the ideality factor, which is a measure of how closely the device follows the ideal p-n junction behaviour.

The methodology of the current work was implemented in two-fold; first, the equivalent circuit was sketched and simulated via the electronic workbench programming of Multisim Power Pro.10. In this simulation approach, the values of *R*_*s*_ and *R*_*p*_ were systematically altered, while the *I-V* characteristics was simultaneously recorded and stored by the grapher view to be exported into the MS excel file. Second, theoretical equations were compiled into the well-known Origin Pro 8 software in order to elucidate the implication of ideality factor (*n*) and cell temperature (*T*).

The saturation current was expressed using the equation reported by Messenger and Ventre [21]:

$$I_{s}=I_{s,ref}{\left[\frac{T_{c}}{T_{c,ref}}\right]}^{3}exp\left[\frac{1}{k}(\frac{E_{g}}{T_{ref}}-\frac{E_{g}}{T_{c}})\right]$$ (2)

Where, *E*_*g*_ is the apparent energy gap of the cell’s active layer, *T*_*c*_ and *T*_*ref*_ are the cell temperature and reference temperature, respectively. The Varshni equation [22] was also utilized to simulate the change of energy gap with temperature as follows:

$$E_{g}(T)=E_{g}(T_{ref})-\frac{AT^{2}}{T+B}$$ (3)

Where, *A* and *B* are fitting parameters and are dependent on the active layer properties. Moreover, the impact of temperature on the light induced current at standard illumination (100 mW/cm^2^) was revealed through the following equation [13]:

$$I_{light}=I_{light}(T_{ref})+\mu (T-T_{ref})$$ (4)

Where, *μ* is the coefficient of temperature dependent light induced current.

## Results and discussion

### Variations in current-voltage (*I-V*) curve

**Fig 2:**
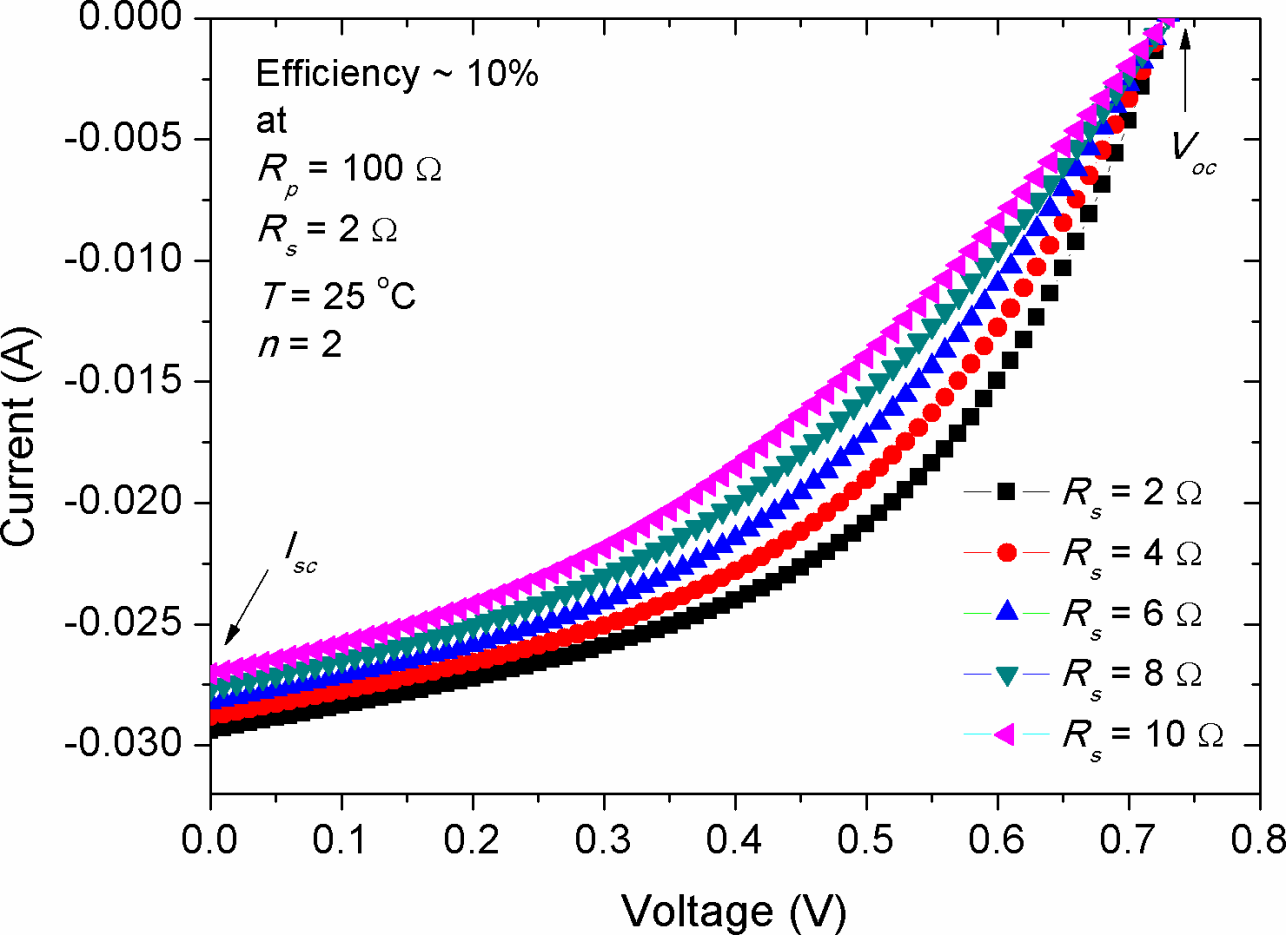
The impact of series resistance on the *I-V* characteristic curve of a simulated solar cell with efficiency of 10%.

Fig 2 shows the shape variation in the *I-V* characteristic of a simulated solar cell with efficiency of 10% at different series resistances (*R*_*s*_). The value of *R*_*s*_ represents the sum of internal resistance, which includes the resistance of active layer and Ohmic contact of the device. It was seen that by increasing *R*_*s*_, no obvious change was happened in the open circuit voltage (*V*_*oc*_). However, the increment in *R*_*s*_ has made a pronounced decrease in the short circuit current (*I*_*sc*_). This was observed to be in agreement with the results reported for inorganic and organic solar cells [23, 24]. The consequence of *I*_*sc*_ variation with the change of *R*_*s*_ for various efficient solar cells is explored later. In practical considerations, the value of *R*_*s*_ for solar panels is usually deteriorated due to the impact of wire connections and aging, leading to unstable photovoltaic performance. Therefore, it is required to keep *R*_*s*_ within a minimal value during the device fabrication and installation in order to enhance the photo-generated current and to achieve the best possible performance. One might think of decreasing the thickness of devices active layer during fabrication, thereby reducing the value of *R*_*s*_ [25]. However, thickness reduction is not always a useful choice, especially for organic solar cells, in which a non-complementary photo-absorption is yielded when the thickness of the devices is kept below 200 nm [26, 27]. Other experimental approaches to reduce *R*_*s*_ can be achieved through a well interplayed donor-acceptor interfaces [28, 29] or by minimizing the contact resistance between the electrodes and active layer [30].

**Fig 3:**
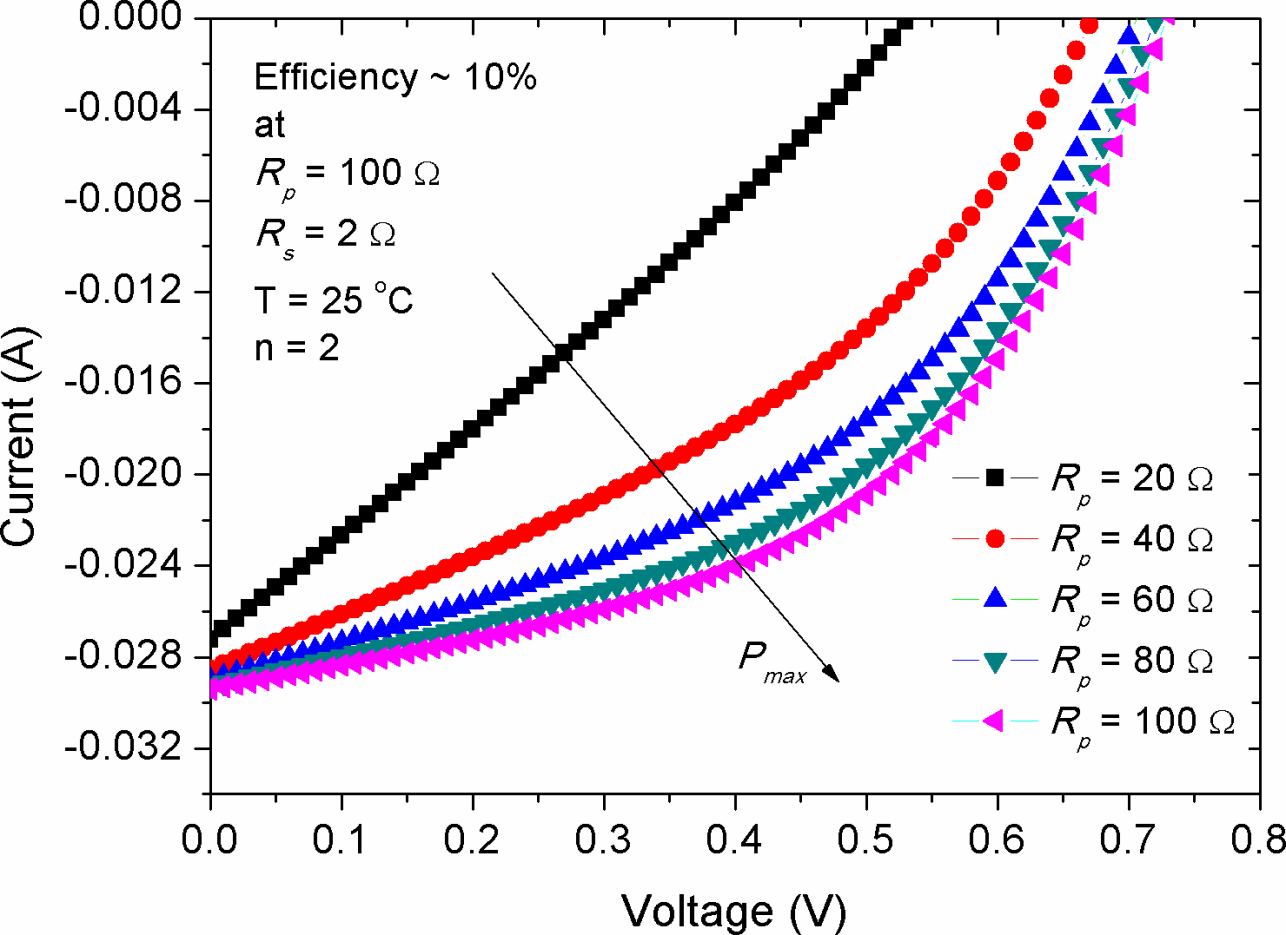
The impact of parallel resistance on the *I-V* characteristic curve of a simulated solar cell having efficiency of 10%.

Another internal electrical parameter affecting the performance of solar panels is the shunt or parallel resistance (*R*_*p*_) of the devices, which takes into account the leakage of current at the donor-acceptor and active layer-electrode boundaries. The value of *R*_*p*_ is usually related to the charge recombination process (either to be geminate or non-geminate), of which the higher recombination rate, in forward bias connection, corresponds to the larger *R*_*p*_ value [31, 32]. In other words, the low carriers’ recombination rate under light illumination, i.e. without biasing, indicates the presence of a large *R*_*p*_ value for the device. Fig 3 shows the effect of *R*_*p*_ alteration on the *I-V* characteristic of a simulated device with 10% efficiency. A close inspection into the figure showed that the increase in *R*_*p*_ has made clear increment in both of *V*_*oc*_ and *I*_*sc*_, while the increment rate of the *V*_*oc*_ was seen to be higher in comparison with that of the *I*_*sc*_. It was practically evidenced that the insertion of PEDOT:PSS layer between the ITO and donor interface in organic solar cells (OSCs) has led to maximizing the value of *V*_*oc*_ [25, 33]. Two theories are accentuated to define the origin of *V*_*oc*_, which are formulated from the difference of valence and conduction band levels between the donor and accepter materials as well as the difference of work functions between the top and bottom electrodes, respectively [34, 35]. Nevertheless, one cannot completely rely on these theories in order to elaborate the origin of *V*_*oc*_ as the change in PEDOT:PSS thickness was seen to alter the value of *V*_*oc*_ [36]. If the two aforementioned theories are fully applicable in OSCs then the impact of PEDOT:PSS thickness on *V*_*oc*_ should not have been pronounced. This is because the level of energy band is not depended on the thickness. Besides, it was found that the difference in work function of the electrodes did not significantly change the value of *V*_*oc*_ [37]. Therefore, the variation in *R*_*p*_ due to charge transfer, emission process and their recombination behaviour [38] might be more impressive to be correlated with the *V*_*oc*_ response.

**Fig 4:**
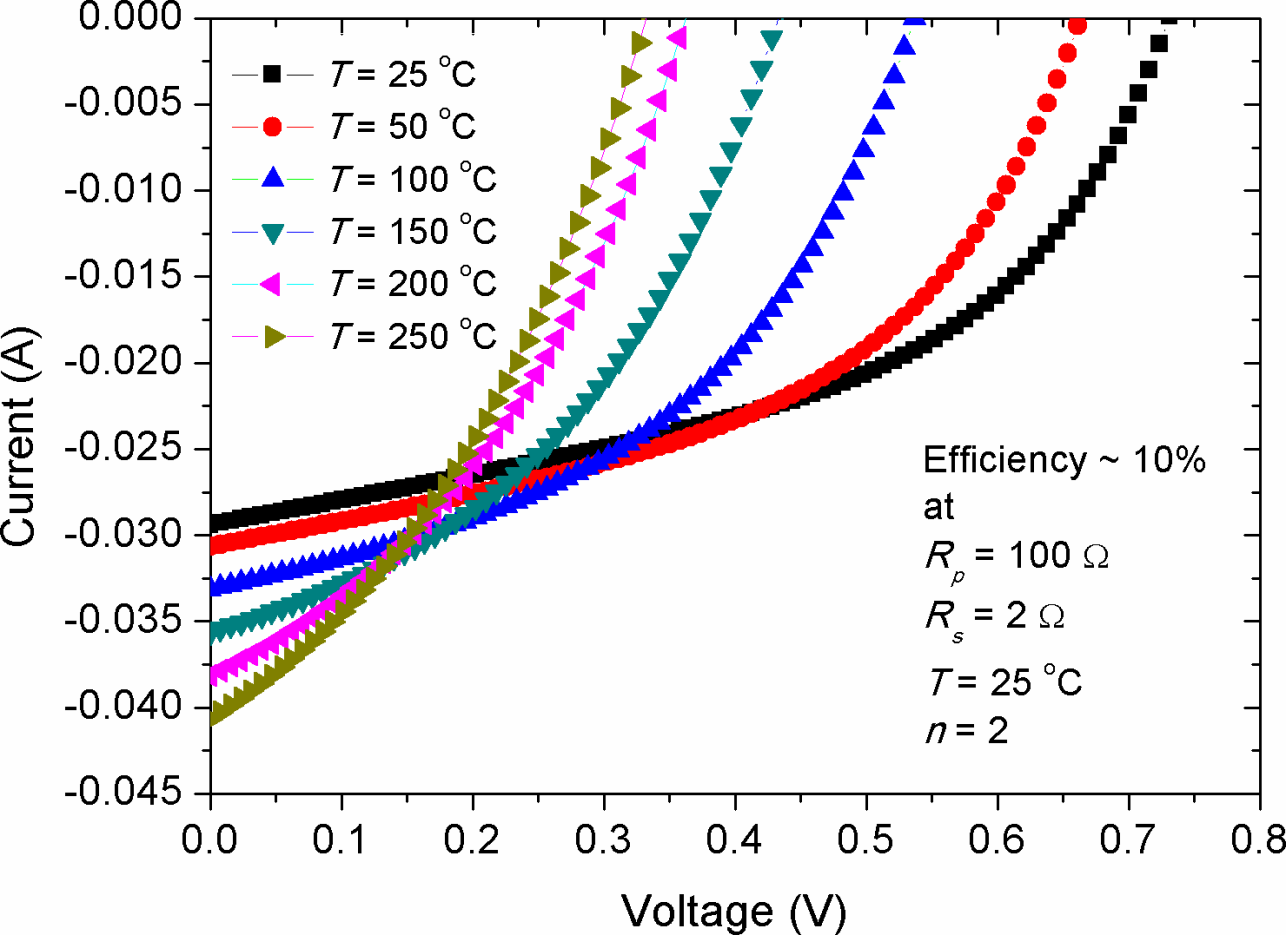
The impact of annealing temperature on the *I-V* characteristics of a simulated solar cell with efficiency of 10%.

Fig 4 shows the variation of *I-V* characteristic due to the change of temperature for a simulated solar cell with efficiency of 10%. The results showed a decrease in *V*_*oc*_ and increase in *I*_*sc*_ upon the rise of solar cells temperature, which is in compliance with the previous theoretical findings [39]. Experimentally, temperature effect produced similar variation trend in the *V*_*oc*_ and *I*_*sc*_ for almost all the types of inorganic solar cells [40–42]. However, this was seen to be different for organic solar cells and the experimental results were in contradiction to that of the simulated ones. For instance, in the small molecular based OSCs [43] and ternary based ones [44], a decrease in the *I*_*sc*_ was observed beyond 80 ^o^C, while in the ternary devices, the value of *V*_*oc*_ stayed relatively unchanged. However, the photovoltaic response in low temperature range, from 25 ^o^C to about 80 ^o^C, was found to be consistent with the simulated results [43, 45]. To conclude, this deviation between simulation and experimental results for organic solar cells requesting the development of a comprehensive model to simulate the temperature impact on these devices.

**Fig 5:**
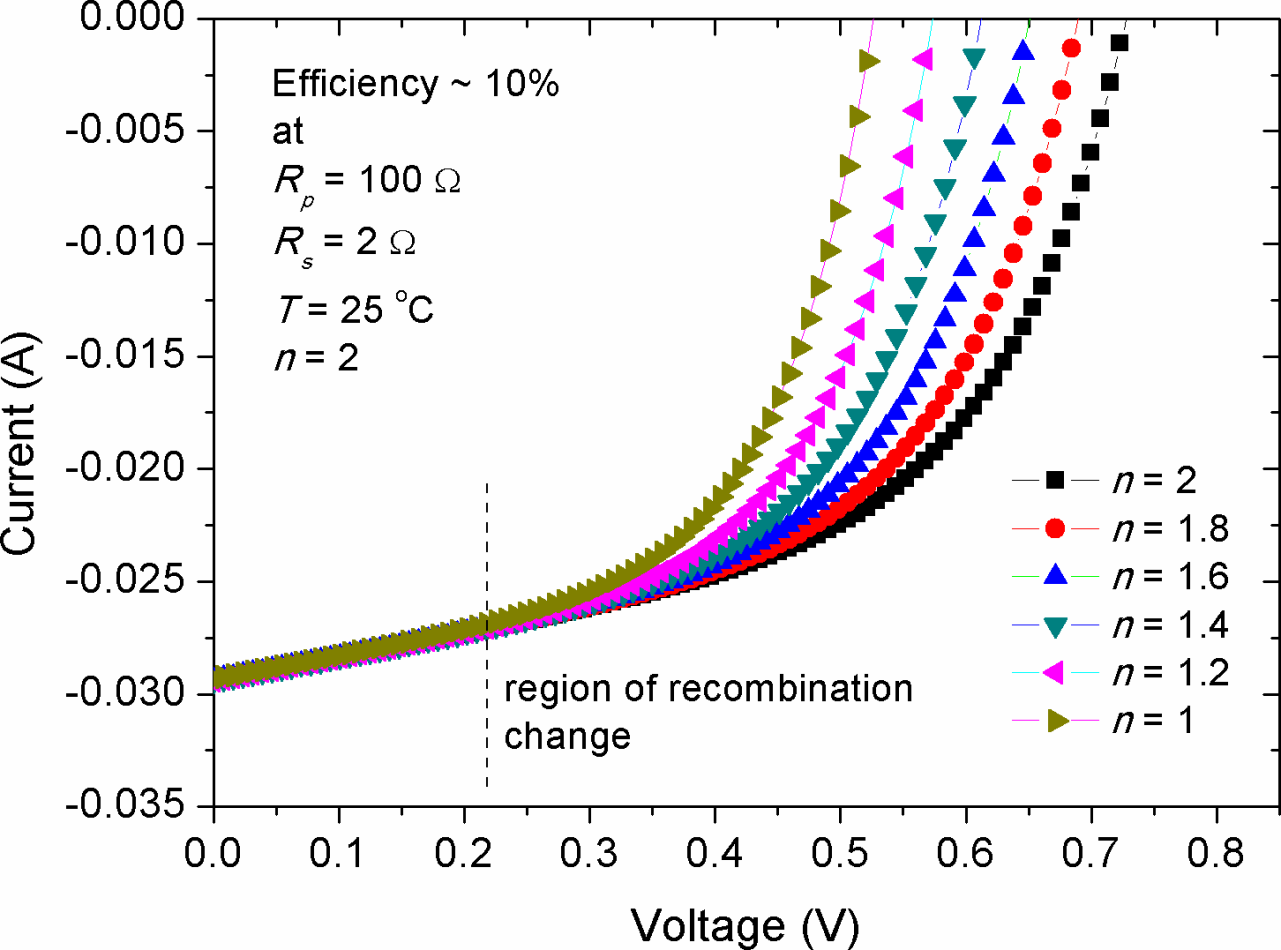
The impact of ideality factor on the *I-V* characteristics of a simulated solar cell with efficiency of 10%.

Fig 5 shows the variation of *I-V* characteristic due to the change of ideality factor (*n*) for a simulated solar cell with efficiency of 10%. The value of *n* is a measure of how closely the device follows ideal p-n junction behaviour, by which valuable information regarding the charge transport and recombination process can be obtained. If the value of *n* = 1, only diffusion currents are flowing in the p-n junction, which corresponds to the band to band recombination process. However, if *n* = 2, the device currents are dominated by charge generation and recombination process, which require states near the middle of interface gap [46, 47]. In the region of large reverse bias, the recombination probability is ideally zero because of the existence of highly sufficient internal electric field to remove barriers against recombination, thereby guarantying a safe reach of free charge carriers to the electrodes. Therefore, it was seen that the *I*_*sc*_ remained relatively unchanged by the increment of ideality factor. However, when the device is forward biased, the major recombination rate is increased, so the photo-generated current is up shifted exponentially. Wetzelaer *et al*. [48] reported that the ideality factor of trap-free solar cells (*n* = 1) is merely governed by bimolecular recombination rather than a trap-assisted recombination. Our numerical results showed that *V*_*oc*_ was significantly affected by the change in ideality factor, in which the value of *V*_*oc*_ was decreased with the reduction of ideality factor. Hence, we might speculate that the ideality factor is directly correlated with *R*_*p*_. This is because the increment in *R*_*p*_ has also contributed in rising *V*_*oc*_ (see Fig 3) and that *R*_*p*_ is inversely proportional to the charge recombination rate [46, 49]. Noteworthy, the simulated result estimated that recombination current at forward bias is higher for the devices with large ideality factor in comparison with those of the low ideality factor.

### Variations in short circuit current (*I*_*sc*_)

**Fig 6:**
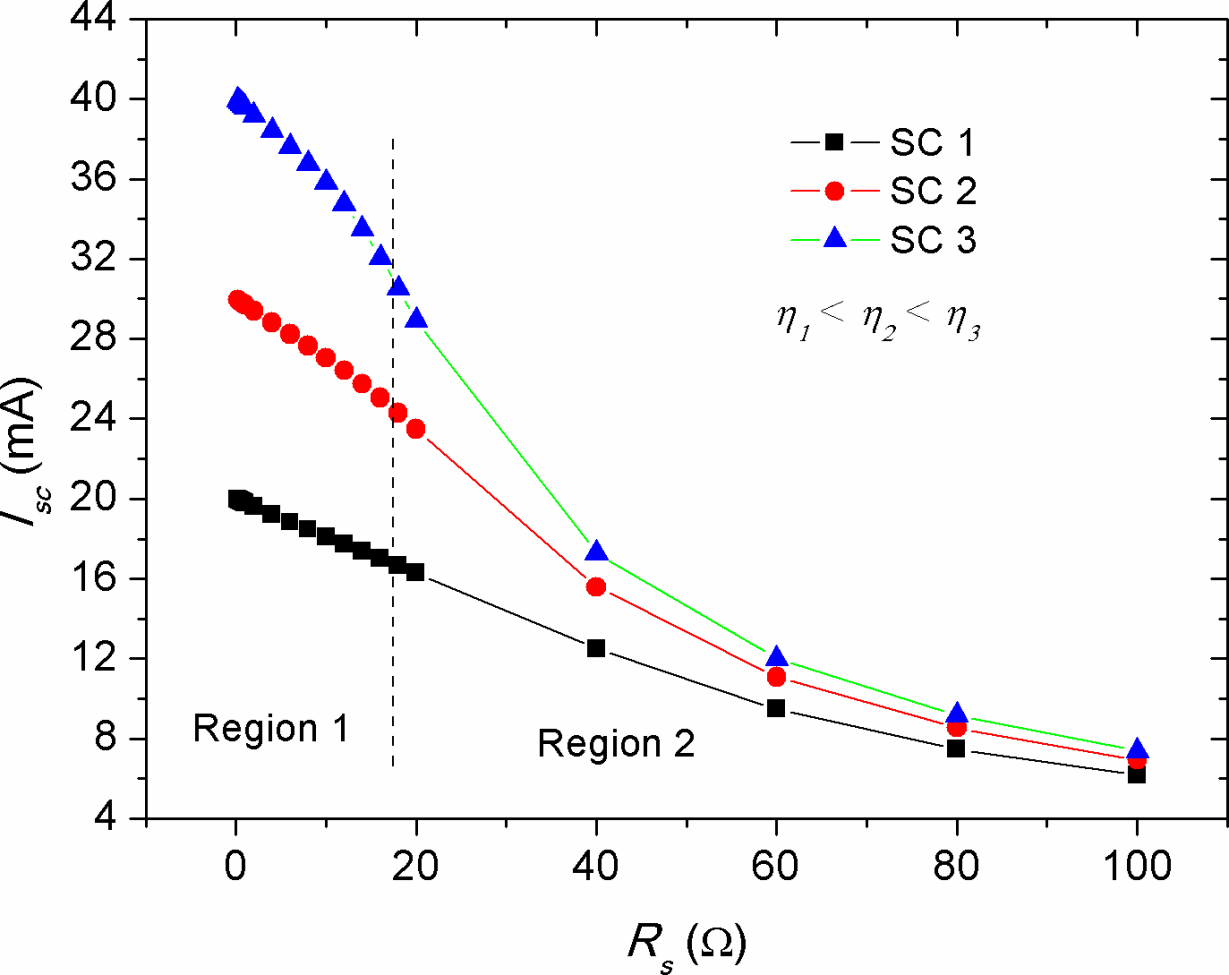
The impact of series resistance (*R*_*s*_) on the short circuit current (*I*_*sc*_) of solar cells with various efficiencies.

**Fig 7:**
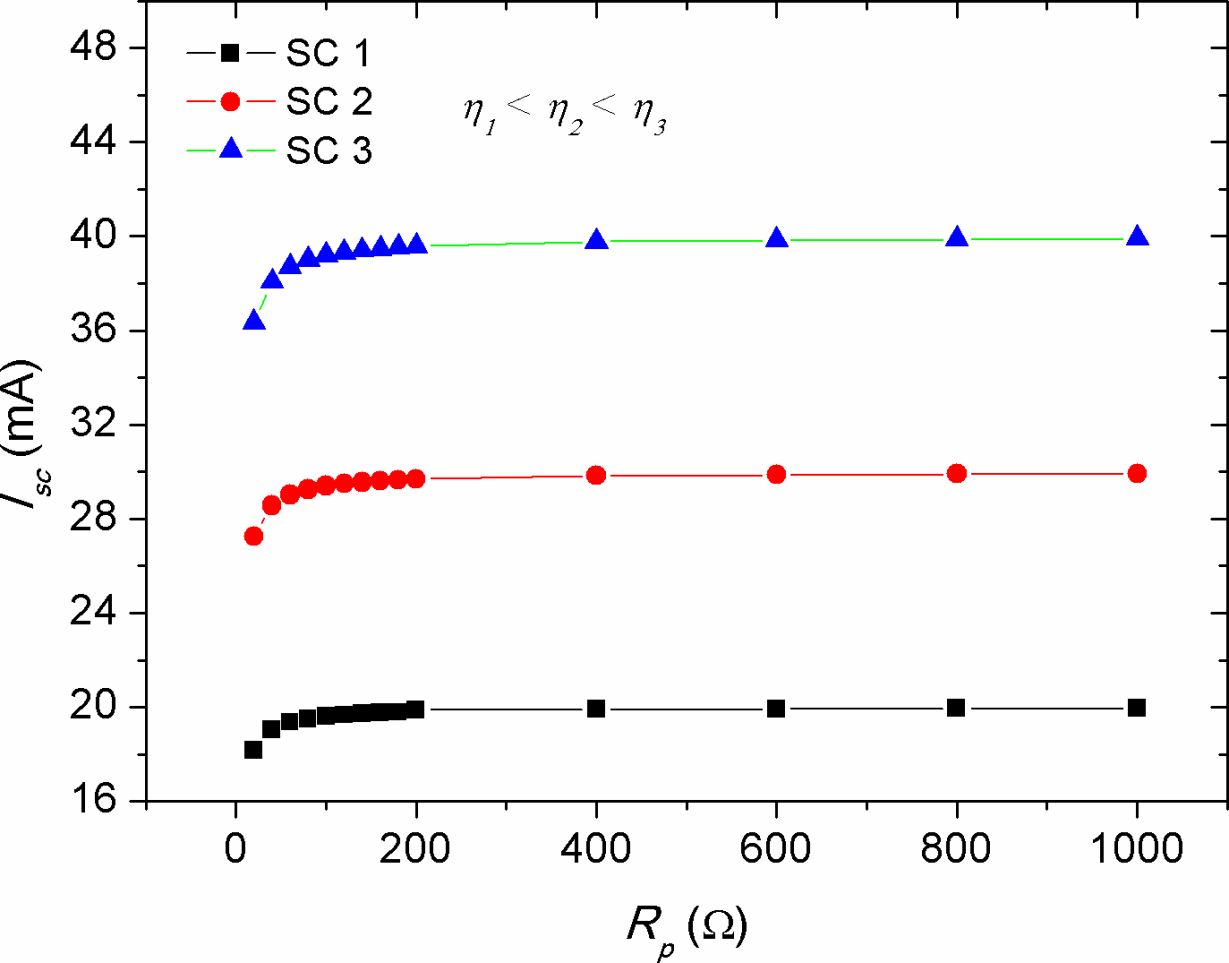
The impact of parallel resistance (*R*_*p*_) on the short circuit current (*I*_*sc*_) of solar cells with various efficiencies.

**Fig 8:**
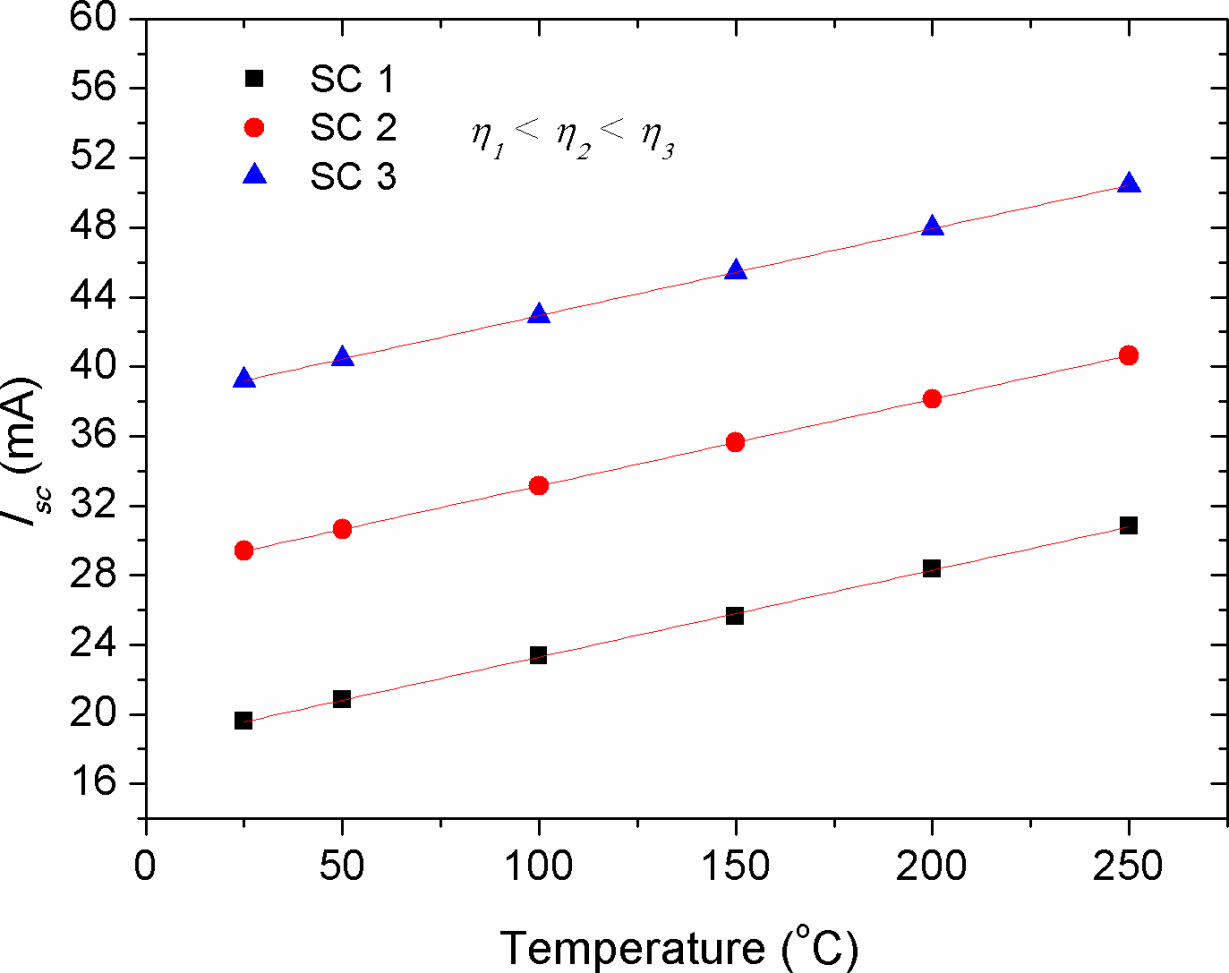
The impact of cell temperature (*T*) on the short circuit current (*I*_*sc*_) of solar cells with various efficiencies.

Figs 6–8 show the effect of *R*_*s*_, *R*_*p*_ and *T* alteration on the *I*_*sc*_ of solar cells (CS)s with different efficiencies, respectively. The manipulation of various efficiencies for the devices in the simulation results was achieved by considering different energy gaps for the active layer materials, namely *E*_*g*_ = 1.2, 1.4 and 1.6 eV. The lower energy gap is resulted in higher photo-generated current, representing solar cells with higher efficiencies in comparison with those of higher energy gap at standard condition. From Fig 6, a linear inverse correlation of *R*_*s*_ with *I*_*sc*_ was seen for various efficient devices in the low resistance range (region 1), while in the high range of *R*_*s*_ (region 2), this correlation showed an exponential decay. The linear region can be elucidated by the presence of a smooth transport of delocalized charge carriers as a result of low bulk resistance, which is activated by the influence of internal electric field. However, the increased bulk resistance promotes accumulated charge carriers between the electrode-active layer interfaces, whereby its role acting upon inducing a non-liner charge injection. Consequently, the highly efficient devices demonstrated a faster deviation from such linearity. It is worth to mention that the negative impact of *R*_*s*_ on the *I*_*sc*_ is stronger in devices with high efficiency in comparison with that of the low efficiency ones. This can be ascribed to the effect of charge transport, which is governed by the space charge limited current (SCLC) [50]. The decrease of *I*_*sc*_ with the increment in *R*_*s*_ can be understood as the internal resistance hindering the drift transport of free charge carriers towards the electrodes under the influence of internal electric field [47]. However, it is known that the increase in active layer thickness of inorganic solar cells is led to increase in *R*_*s*_, the application of a thin layer of bathocuproine between Al electrode and active layer in OSCs has improved *I*_*sc*_ [51]. Hence, it is evidenced that the increase in active layer thickness of OSCs does not always bring a negative impact on *I*_*sc*_. This is happened because of the enhanced donor-acceptor interface between the moieties constituents, which in turn facilitates efficient transport of free charge carriers. In contrast to the negative impact of *R*_*s*_ on the *I*_*sc*_, the effect of *R*_*p*_ was seen to be positive and showed a reverse trend (see Fig 7). Interestingly, the achievement of a relatively low practical *R*_*p*_ of about 200 Ω is enough to obtain a stable photo-current generation and deliver optimum *I*_*sc*_, provided that the series resistance of the cell is kept below 2 Ω. The value of *R*_*s*_ and *R*_*p*_ are regarded to be internal factors influencing the performance of solar cells, while temperature variations of the cell is considered as an external influence due to ambient temperature change or thermal annealing process. Fig 8 depicts the effect of cell temperature (*T*) on the *I*_*sc*_, in which a pronounced increase in the *I*_*sc*_ was noticed versus temperature. This was occurred because of the enhanced thermal excitation of charge carriers, thereby reducing the active layer’s energy gap and hence promoting charge carriers transport. Noteworthy, the increment rate of *I*_*sc*_ with temperature was theoretically found to be constant for solar cells with different efficiencies (see Fig 8), implying that the temperature impact on *I*_*sc*_ is an energy gap independent phenomenon. However, the estimation of linearly increased *I*_*sc*_ with *T* can only be applicable for inorganic solar cells, while for OSCs this was deviated from experimental evidences, as it was discussed previously in Fig 4.

### Variations in open circuit voltage (*V*_*oc*_)

**Fig 9:**
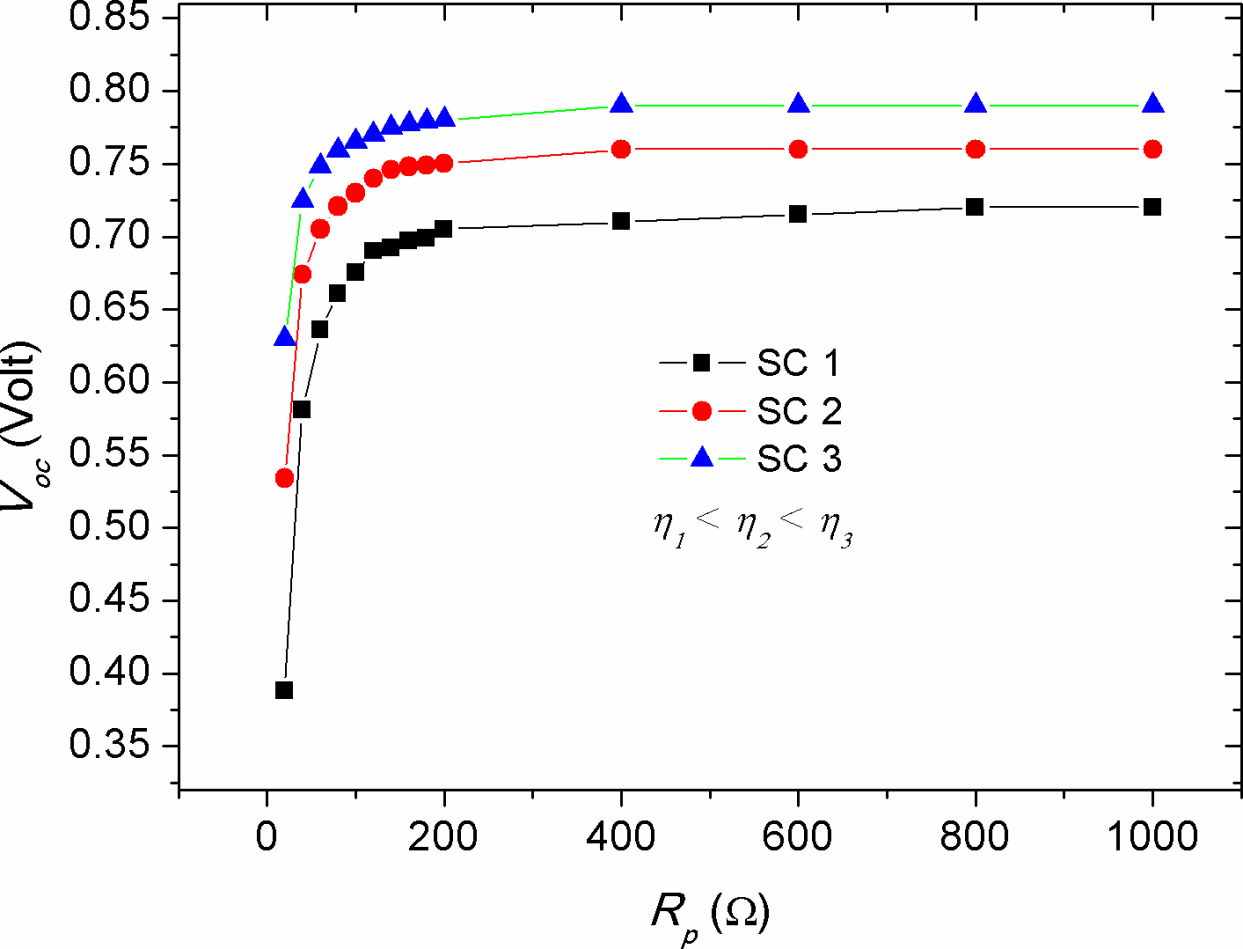
The impact of parallel resistance (*R*_*p*_) on the open circuit voltage (*V*_*oc*_) of solar cells with various efficiencies.

**Fig 10:**
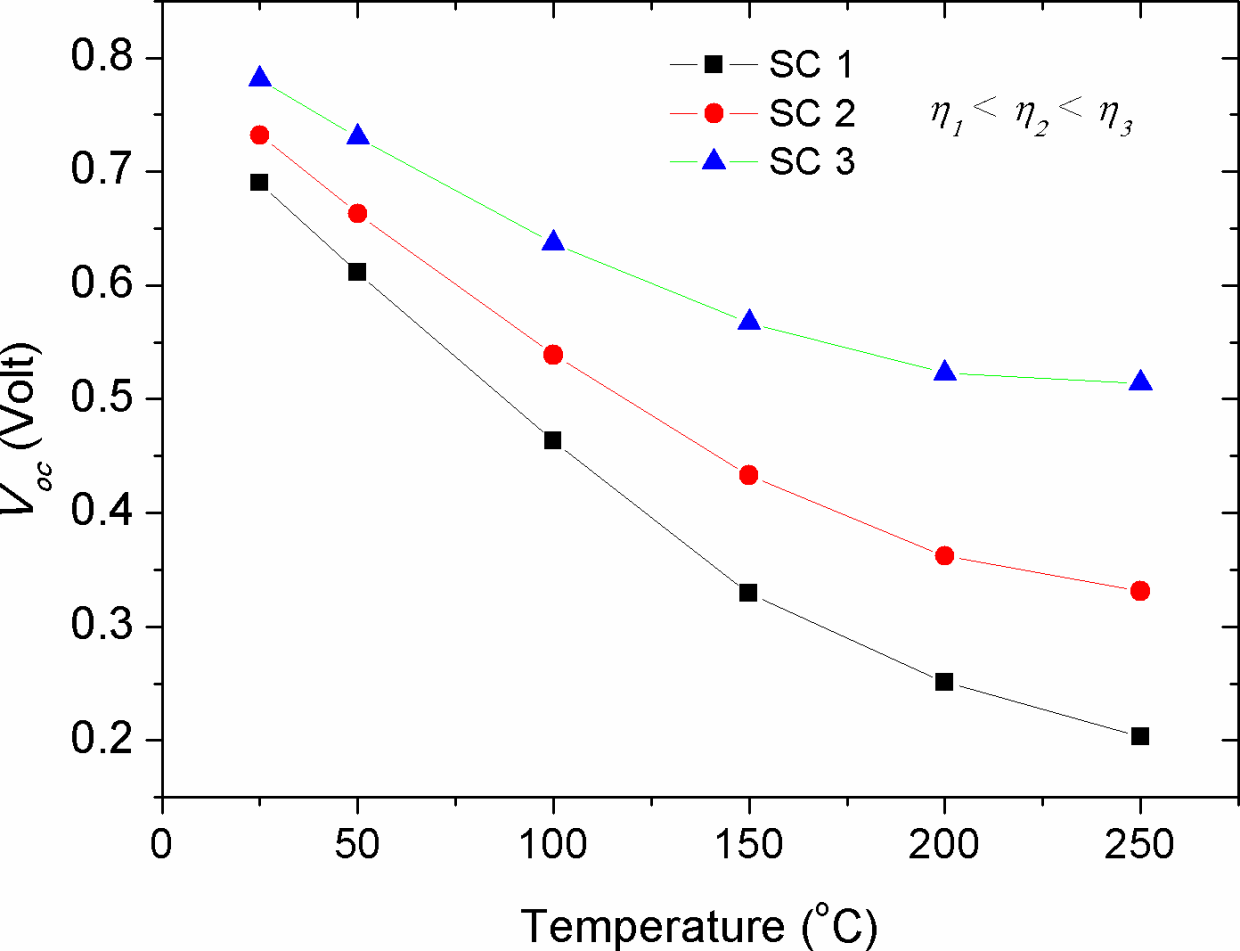
The impact of cell temperature (*T*) on the open circuit voltage (*V*_*oc*_) of solar cells with various efficiencies.

**Fig 11:**
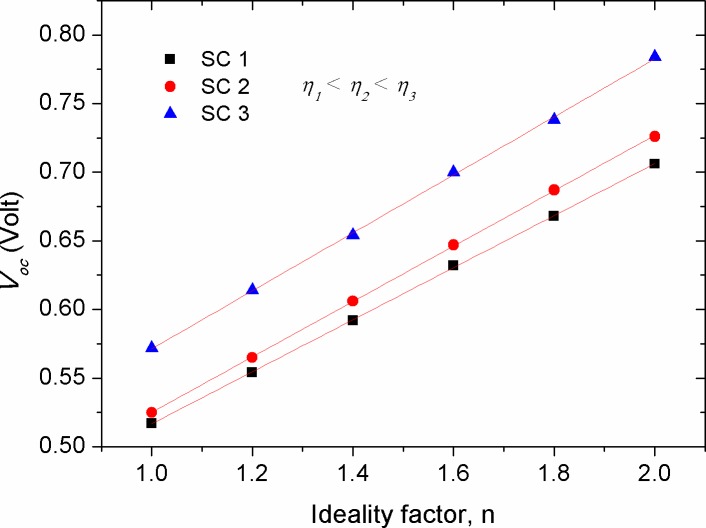
The impact of ideality factor (*n*) on the open circuit voltage (*V*_*oc*_) of solar cells with various efficiencies.

Figs 9–11 show the effect of *R*_*p*_, *T*, and *n* parameters on the *V*_*oc*_ of solar cells with different efficiencies, respectively. One can notice that *V*_*oc*_ is enlarged with the increase of *R*_*p*_ to appoint where a plateau region is observed beyond *R*_*p*_ = 200 Ω. The rise of *V*_*oc*_ with *R*_*p*_ represents an increased potential barrier of the diode, at which the charge recombination process requires higher forward voltage to set off the photo-generated current under illumination in comparison with that of the low *R*_*p*_ devices. As *R*_*p*_ is related to the charge recombination process [31, 32], the complete saturation of *V*_*oc*_ above *R*_*p*_ = 800 Ω indicating that *V*_*oc*_ is limited by the recombination rate (the number of recombined charge carriers per time). Noticeably, similar recombination rate is speculated for devices with various efficiencies as long as they attain high enough parallel resistance of about *R*_*p*_ = 800 Ω. In support to the simulated results, experimental evidences showed that interplaying donor-acceptor ratio did not produce any obvious change in the *V*_*oc*_ despite that the variation in *R*_*p*_ was appeared at values greater than 14.5 kΩ (290 Ω.cm2) [52]. As such, the impact of *R*_*p*_ on the *V*_*oc*_ can be ruled out at *R*_*p*_ equals or greater than a threshold value of 800 Ω. Hence, during the fabrication process of solar cells special attention need to be paid in order to locate the parallel resistance beyond the threshold value. Furthermore, it was seen that the increased ratio of *R*_*p*_*/R*_*s*_ did not change the *V*_*oc*_ trend, but has made *I*_*sc*_ to become constant faster. Based on the equation of single-diode model, *V*_*oc*_ can be determined when the forward biased current is set to zero (*I* = 0). Hence, the expression for open circuit voltage is obtained from the explicit equation:

$$I_{s}\times \exp\left(\frac{qV_{oc}}{nK_{B}T}\right)+\frac{V_{oc}}{R_{p}}=I_{light(open)}+I_{s}$$ (5)

Where, *I*_*light(open)*_ is the photo-generated current at open circuit condition. By having a high value of *R*_*p*_ in Eq 5, the second term on the left side of the equation can be neglected and *V*_*oc*_ is no more dependable on *R*_*p*_,

$$V_{oc}=\frac{nK_{B}T}{q}\times ln\left[\frac{I_{light(open)}}{I_{s}}+1\right]$$ (6)

Eq 6 demonstrates that the higher *V*_*oc*_ is related to the more efficient solar cell, as can be seen in Fig 9. On the other hand, a logarithmic decay in *V*_*oc*_ with temperature (*T*) was seen (see Fig 10), which is inconsistent with Eq 6 in the sense that *T* is linearly proportional with *V*_*oc*_. This discrepancy can be concealed that *I*_*s*_ is enlarged with the increase of *T*, thereby decreasing the value of *V*_*oc*_. Noteworthy, the results of Fig 10 show that the impact of temperature on *V*_*oc*_ is less pronounced in the devices with high efficiency. Another parameter affecting *V*_*oc*_ is ideality factor (*n*), as shown in Fig 11. Our numerical results showed that *V*_*oc*_ is significantly affected by the change of ideality factor, in which the value of *V*_*oc*_ is increased with the increase of ideality factor.

### Variations in fill factor (*FF*)

The fill factor (*FF*) of a solar cell is defined by the ratio of maximum power (*P*_*max*_ = *I*_*max*_ × *V*_*max*_), which is capable to be delivered to a load to that theoretically produced by the cell (*I*_*sc*_ × *V*_*oc*_),

$$FF=\frac{I_{max}\times V_{max}}{I_{sc}\times V_{oc}}$$ (7)

It plays important role in controlling the efficiency of the cell and represents how easily the photo-generated carriers are extracted out of the cell. The shape of *I-V* curve can be a straightforward indication on how close to ideal the devices are fabricated, as the better the device performance the closer the *I-V* shape to a rectangle. Because *FF* is intricately influenced by many factors, it is the least understood parameter in solar cells. It has been claimed that the *FF* related shape of *I-V* curve is depended on the interface quality between the active layer and negative electrode. The S-kink shape in the fourth quadrant of *I-V* curve is almost due to a poor contact formation during the deposition of cathode electrode, which ultimately leads to unbalanced mobility of electron and hole and low minor surface recombination [46]. Therefore, a slow rate deposition of cathode electrode is practically requested to overcome these shortcomings. Recalling Fig 2, one can see that the *I-V* shape is convex, indicating the existence of small Ohmic contact between the cathode electrode and active layer. Consequently, the concave shape of *I-V* curve is expected to appear when there is a high barrier between the cathode electrode and active layer, by which charge accumulation occurs and inefficient exciton dissociation is obtained. The non-linear decrease of *FF* with the increase of *R*_*s*_ can be expressed by the following empirical equation [33]:

$$FF={FF}_{ref}\left(1-R_{s}\frac{I_{sc}}{V_{oc}}\right)$$ (8)

**Fig 12:**
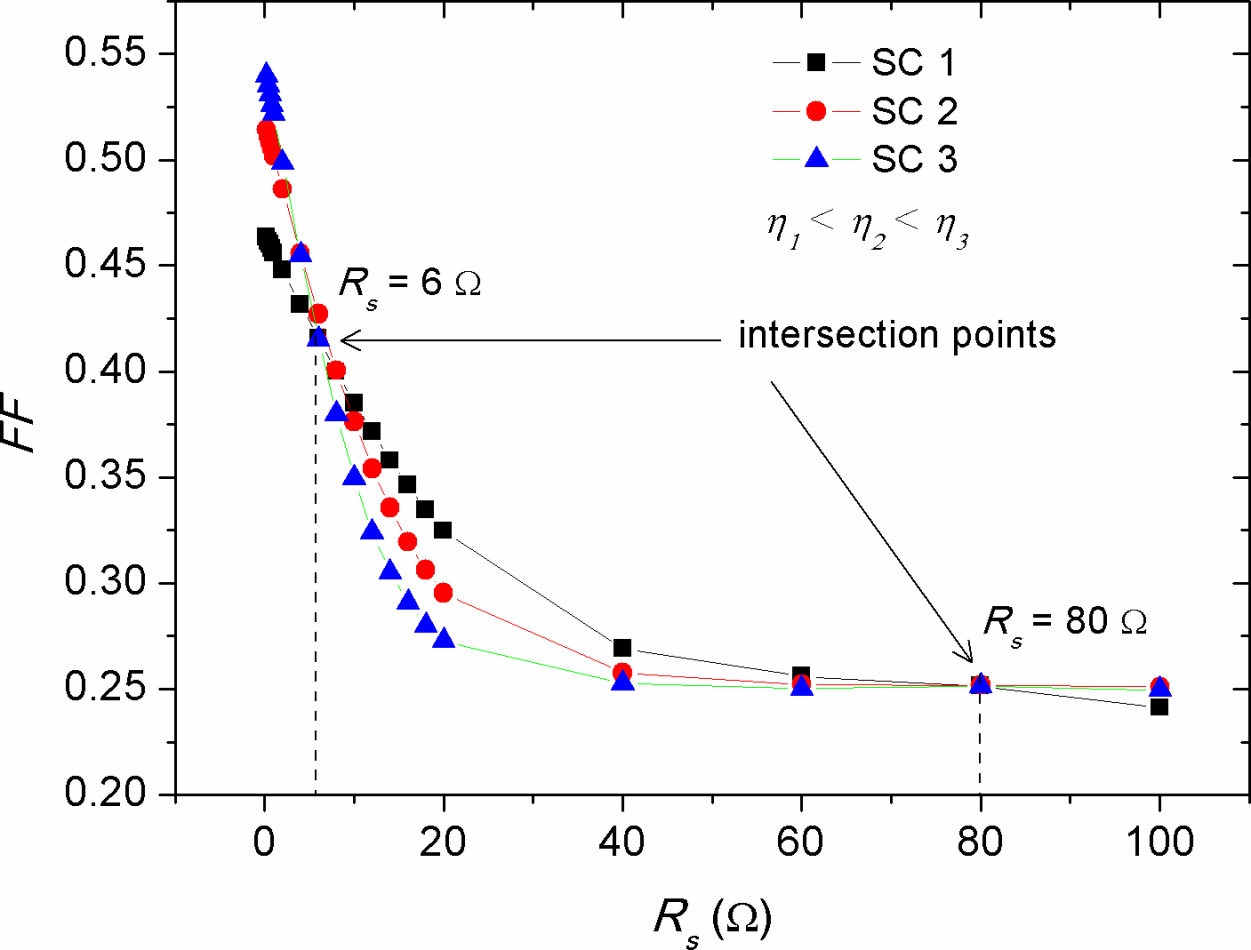
The impact of series resistance (*R*_*s*_) on the fill factor (*FF*) of solar cells with various efficiencies.

**Fig 13:**
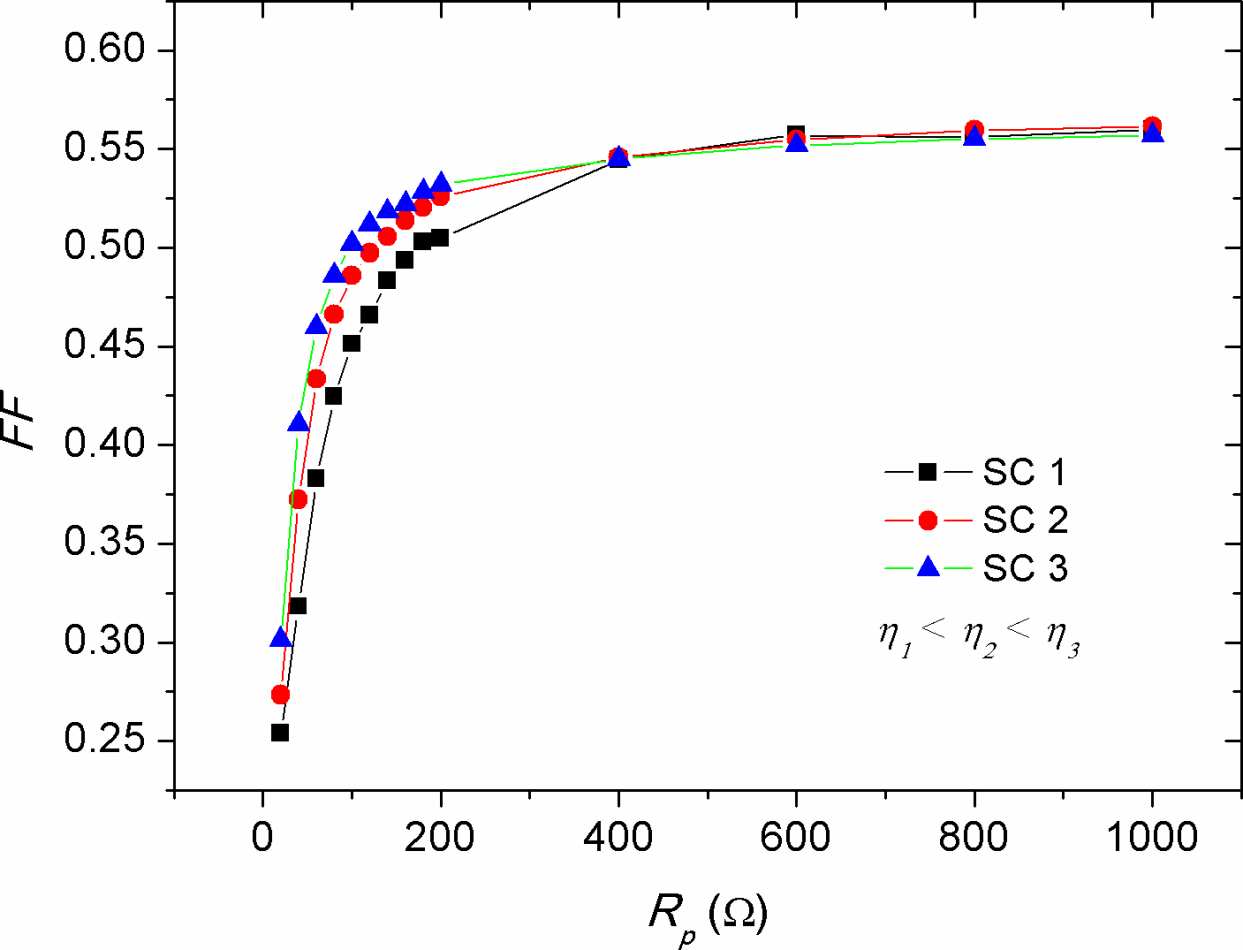
The impact of parallel resistance (*R*_*p*_) on the fill factor (*FF*) of solar cells with various efficiencies.

Where, *FF*_*ref*_ is the reference fill factor of the solar cell at a defined *R*_*s*_. Fig 12 shows the reduction of *FF* against the increment of *R*_*s*_ for solar cells with various efficiencies. There have been two points correspond to the *R*_*s*_ of 6 Ω and 80 Ω, respectively, at which the *FFs* of the devices are equal. This *FF* similarity indicated the presence of a shape consistency in the *I-V* curve regardless of the efficiency of the devices. Comparably, Eq 8 illustrates that the decrease rate in *FF* for highly efficient solar cells should be greater than that of the low efficient one (see Fig 12), which is again in agreement with the fact that the impact of *R*_*s*_ on *I*_*sc*_ is more pronounced in solar cells with high efficiencies, as was shown in Fig 6. In contrast to that of the *R*_*s*_ effect, the increase in *R*_*p*_ was found to produce a non-linear increase in the *FF*, as shown in Fig 13. By considering the results of Figs 7 and 8, in which the values of *I*_*sc*_ and *V*_*oc*_ were increased with the rise of *R*_*p*_, and upon the utilization of Eq 7, one can speculate that the *FF* should have been decreased with the increase of *R*_*p*_ as a result of increasing *V*_*oc*_. But this was seen not to be happened, elucidating that the increase of *R*_*p*_ has made changes in the *I-V* shape (see Fig 3) so that the value of maximum power point (*P*_*max*_) is become high. Consequently, the rapid rise in *FF* versus *R*_*p*_ is attributed to the weakened recombination rate of majority charge carriers, while that the internal electric field inducing the exciton dissociation is getting saturated with further increase of *R*_*p*_.

**Fig 14:**
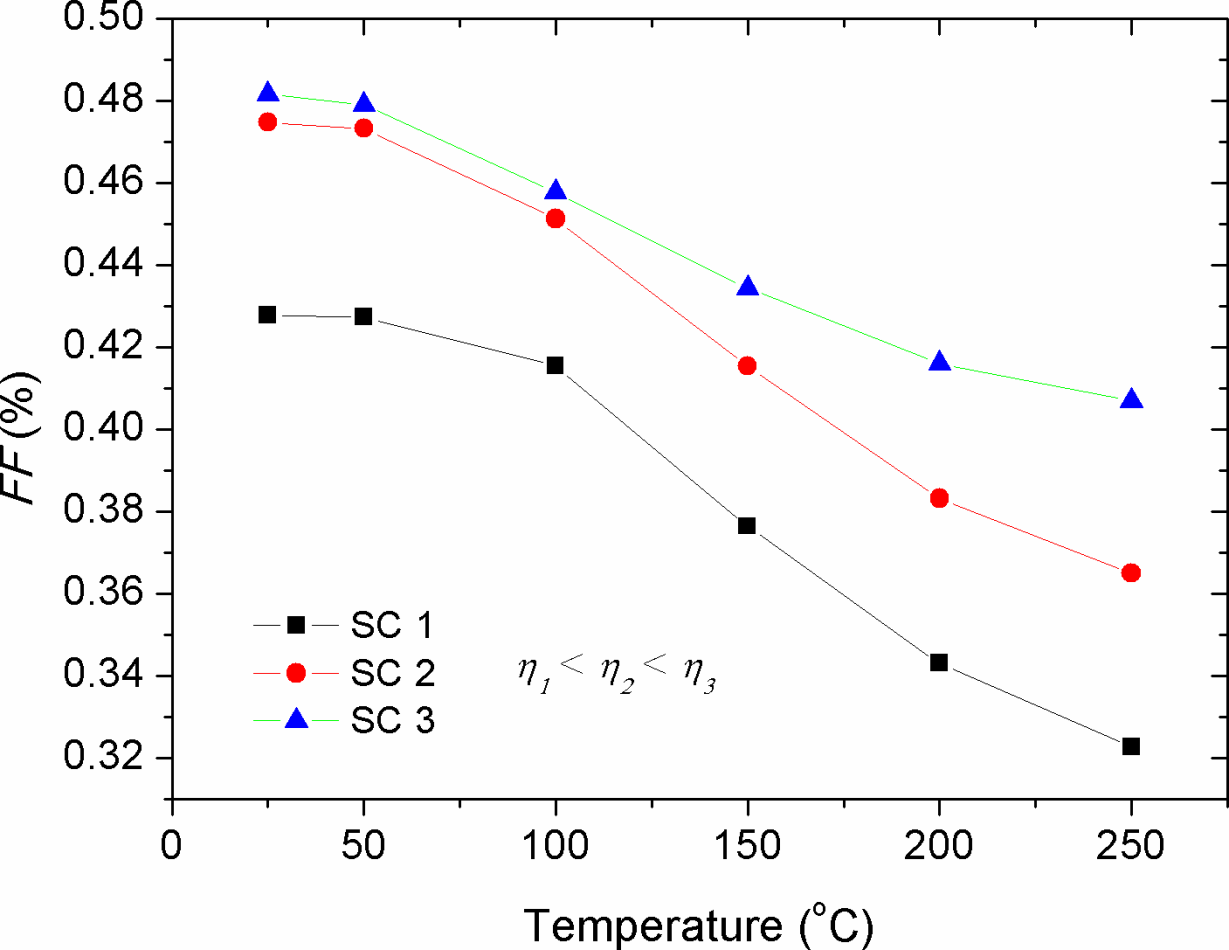
The impact of cell temperature (*T*) on the fill factor (*FF*) of solar cells with various efficiencies.

**Fig 15:**
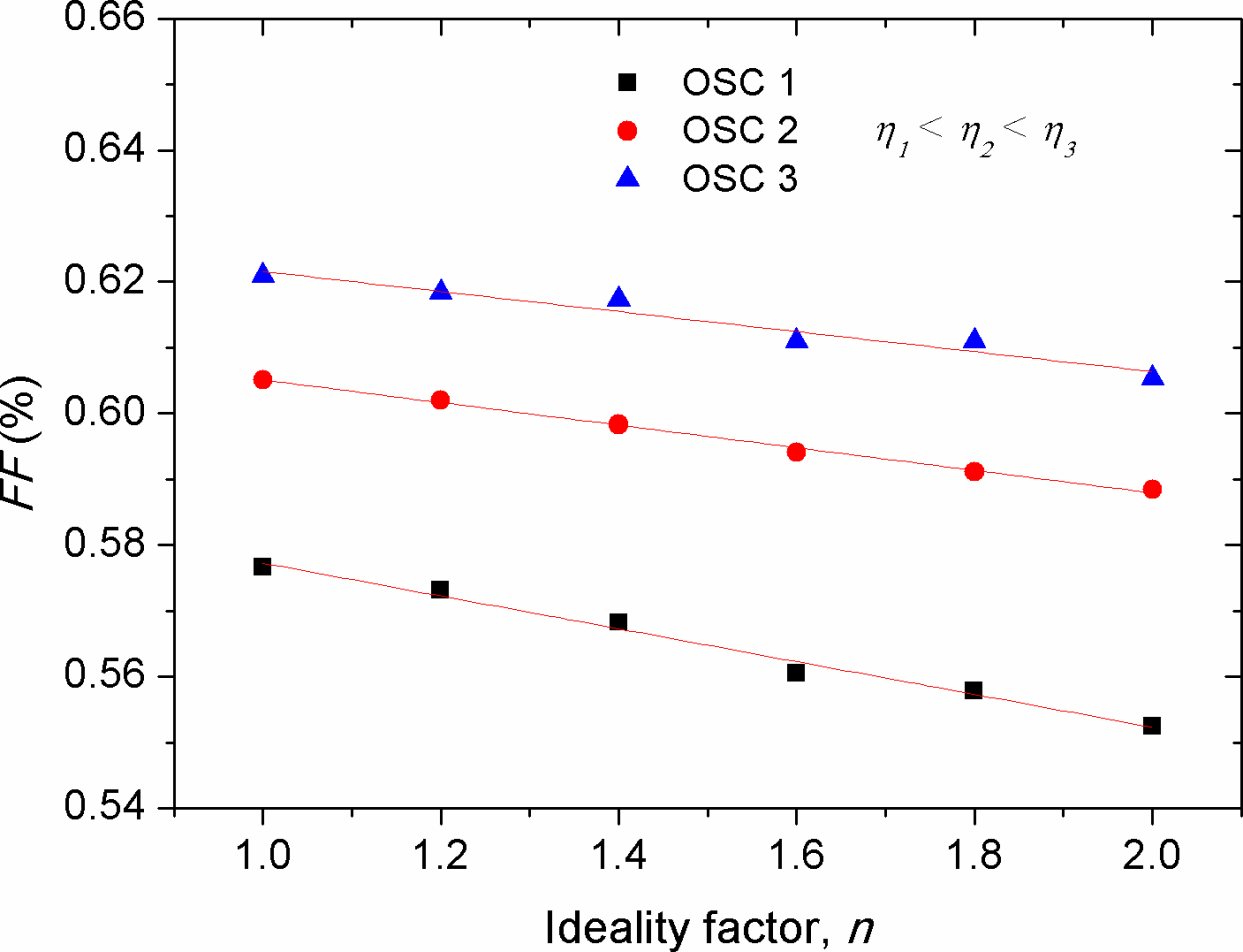
The impact of ideality factor (*n*) on the fill factor (*FF*) of solar cells with various efficiencies.

Fig 14 shows the effect of cell temperature (*T*) on the fill factor of solar cells with various efficiencies. Overall, the increase in *T* depicted a decrease in *FF*, with its effect being more pronounced in the low efficiency devices compared with that of the high efficiency ones. Because of the complexity of *FF* dependence on the internal and external parameters affecting solar cells, there is not a generalized equation up to date by which the impact of *T* on the *FF* is being revealed. However, in its simple form, one can elucidate that the decreased *FF* against *T* is due to the change in the convexity shape of the *I-V* curve, in which the increment rate of *I*_*sc*_ is more than the decrement rate of *V*_*oc*_ so that Eq 8 can be safely held. Comparably, experimental results showed a non-monotonic change in the *FF* with the change of *T* [43, 53], indicating that the effect of *T* on the *FF* is complex, leading to a simultaneous tuning of the internal parameters such as *R*_*s*_, *R*_*p*_ and *n*. Therefore, the solar cells whose temperature effect has resulted in the decrease of their *R*_*s*_ demonstrated enlarged *FF* upon thermal annealing. As shown in Fig 15, the increase in ideality factor produced a linear decrease in *FF*, with its stronger effect on the low efficiency devices. Since *FF* is a geometrical related parameter of *I-V* curve in the forward bias, we can conclude that the prevalence of the free charges recombination over their extraction is the major cause behind such decrement in the *FF*.

### Variations in efficiency (*η*)

The power conversion efficiency (*η*) of solar cells is defined by the ratio of maximum electrical power (*P*_*max*_) to the optical power (*P*_*in*_) of incident photons:

$$ɳ=\frac{P_{max}}{P_{in}}=\frac{FF\times I_{sc}V_{oc}}{P_{in}}$$ (9)

**Fig 16:**
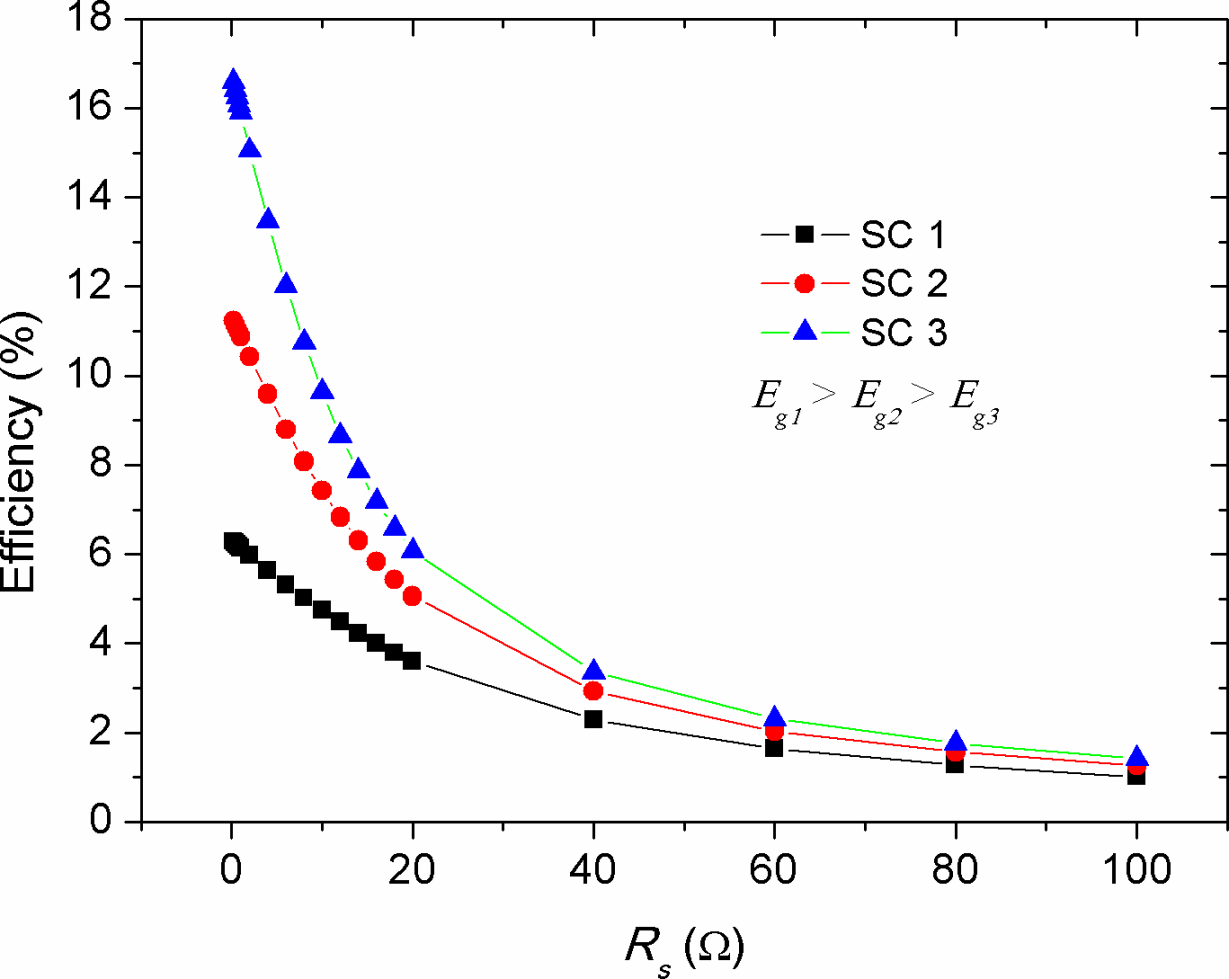
The impact of series resistance (*R*_*s*_) on the efficiency (*η*) of solar cells with various energy gaps of their active layer.

**Fig 17:**
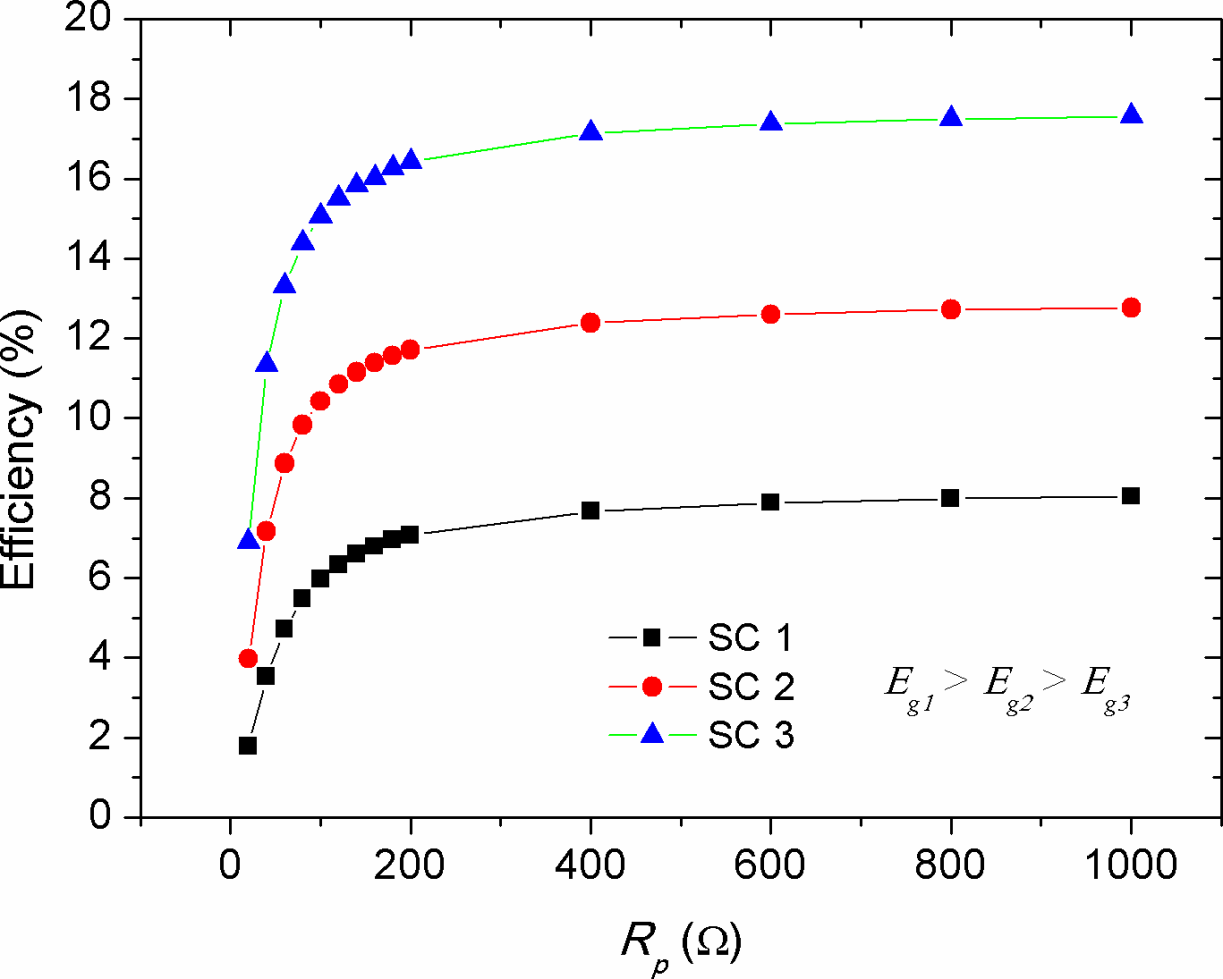
The impact of parallel resistance (*R*_*p*_) on the efficiency (*η*) of solar cells with various energy gaps of their active layer.

**Fig 18:**
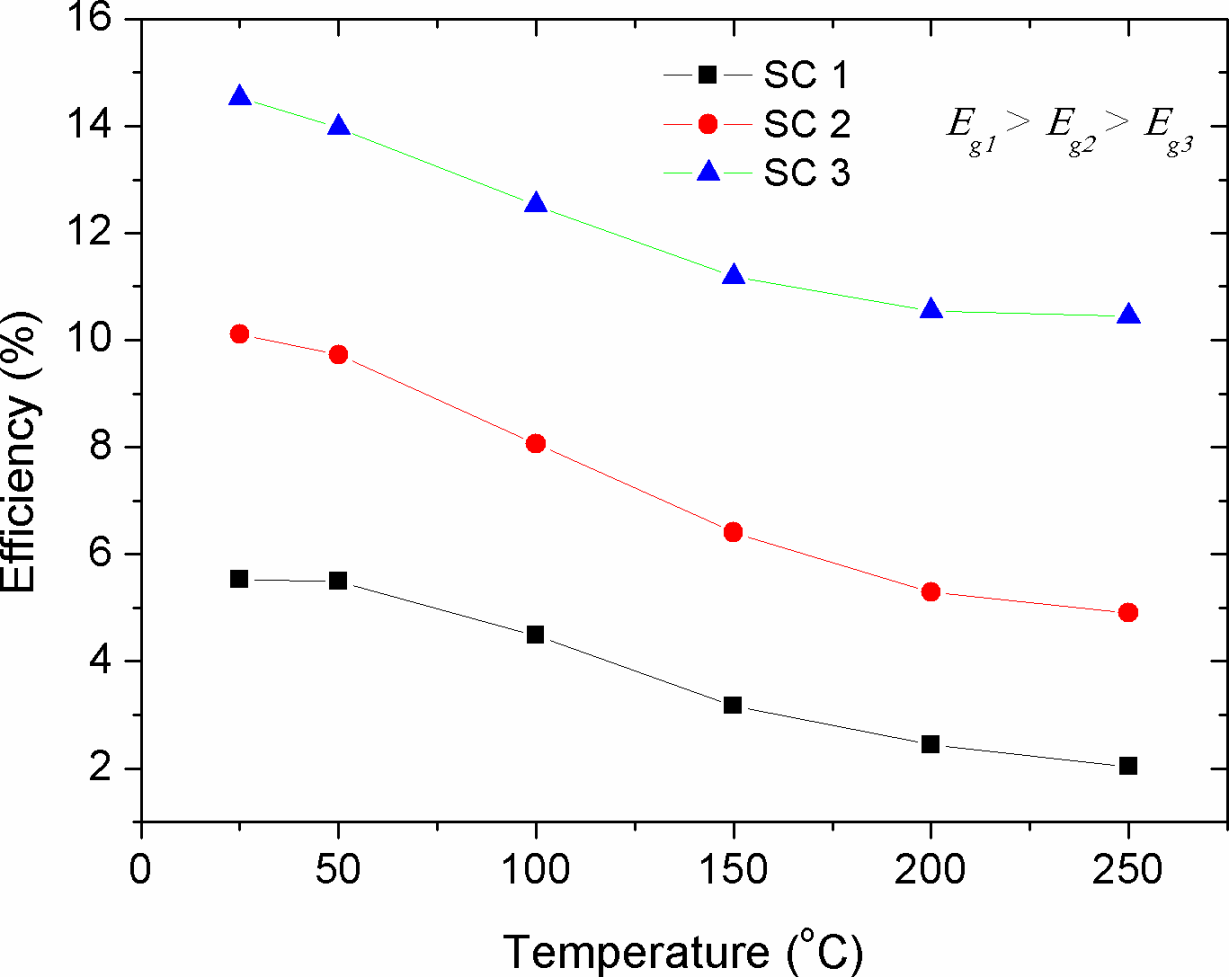
The impact of cell temperature (*T*) on the efficiency (*η*) of solar cells with various energy gaps of their active layer.

**Fig 19:**
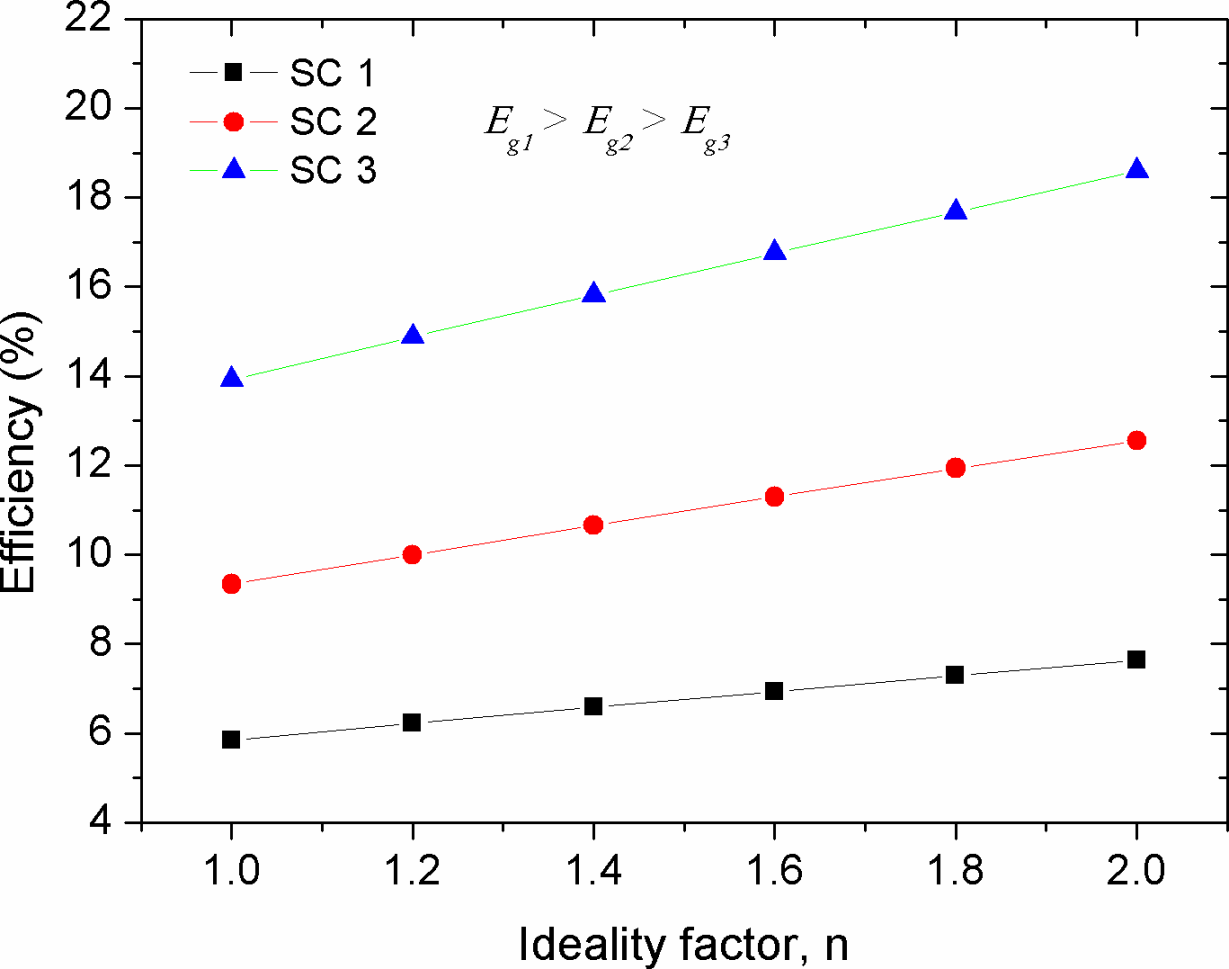
The impact of ideality factor (*n*) on the efficiency (*η*) of solar cells with various energy gaps of their active layer.

Figs 16–19 show the theoretical impact of *R*_*s*_, *R*_*p*_, *T* and *n* on the efficiency of solar cells with different energy gaps of their active layers, respectively. The *R*_*s*_ and *R*_*p*_ parameters are generally correlated with the physical properties of the active layer and the architectural formation of the device. The increase in *R*_*s*_ was led to exponential decrease in efficiency, as shown in Fig 18, with its stronger detrimental impact on the devices with lower energy gap (higher efficiency). However, *R*_*p*_ increment has caused a positive exponential increase in the efficiency of the cells (see Fig 17). Therefore, in practical point of view, researchers should be curious about tuning the structure and morphology of the devices active layers in order to reduce the value of *R*_*s*_ and maximizing *R*_*p*_. Fig 18 shows a general trend of decreasing efficiency of solar cell devices against temperature. This was found to be in agreement with the experimental investigations [41, 54]. The decrease in efficiency with temperature is attributed to increased internal carrier recombination rates, resulted from increased carrier concentrations. It can be seen from the simulated results that a low temperature profile of about 50 ^o^C might have a positive impact on the efficiency enhancement of low performance devices. This was also found to be experimentally true for the OSCs operated at relatively low elevated ambient temperature of about 55 ^o^C [55]. Therefore, it can be concluded that moderate temperature rise in the devices with high energy gap of their active layers can help improving the efficiency, while high temperatures are acted upon deteriorating the efficiency and decreasing the devices performance [56, 57]. Fig 19 shows that the increase in ideality factor of the active layers has caused a linear increase in the devices efficiency. Despite an obvious decrease in the *FF* with the increase of the ideality factor (see Fig 15), the efficiency of the solar cells was yet enhanced. This can be concealed that the increase in ideality factor has caused a reasonable increase in *V*_*oc*_, thereby improving the power conversion efficiency of the devices based on Eq 9. It is worth mentioning that when a real photovoltaic module is simulated, the ideality factor of the series connected cells should be summed up [13]. Therefore, in such a case the increment in the ideality factor is counted for the increased number of string cells, which by then the efficiency of the solar module is expected to be remained constant [58]. In our discussion, the ideality factor variation, shown in Fig 19, is considered to be due to other factors such as fabrication process, aging and temperature change for a single compact solar cell or solar module. Noteworthy, the exponential correlations of PV panel efficiency with enlarged internal resistance and temperature elevation, shown in Figs 16 and 18, can be interestingly included into the existed models [18, 19] when an improved optimization of energy management and power smoothing of solar PV systems are targeted. This is because the optimized PV plant is complicated by oscillating PV output due to uncertain environmental conditions, internal resistance variations and aging.

## Conclusions

The single-diode modelling approach was employed to correlate the parasitic resistances, temperature and ideality factor with the photovoltaic parameters of solar cells. The impact of series resistance (*R*_*s*_) was seen to be detrimental on the performance of the devices, especially on the devices with small energy gap of their active layers. On the contrary, an increased parallel resistance (*R*_*p*_) is practically requested because of its positive impact on rising *V*_*oc*_ and *I*_*sc*_ of the devices. The achievement of *R*_*p*_ = 800 Ω was found to be enough to produce a stable *I*_*sc*_ and *V*_*oc*_ (S1 Table). Noteworthy, the increment rate of *I*_*sc*_ with temperature was theoretically found to be constant for solar cell devices with different efficiencies, implying that the temperature impact on *I*_*sc*_ is an energy gap independent process. Our numerical results showed that *V*_*oc*_ is decreased with the reduction of ideality factor and increased temperature, while *I*_*sc*_ remained relatively unchanged by the increase of ideality factor. The rapid rise in *FF* versus *R*_*p*_ was attributed to the weakened recombination rate, while the internal electric field inducing the exciton dissociation is become saturated with further increase of *R*_*p*_. Because of the complexity of *FF* dependence on the internal and external parameters affecting solar cells, there is no a generalized equation up to date, by which the impact of *T* on the *FF* is being revealed. Comparably, the experimental evidences showed a non-monotonic decrease in the *FF* upon thermal annealing, indicating that the effect of *T* on the *FF* was indirect and was made through the tuning of the internal features such as *R*_*s*_, *R*_*p*_ and ideality factor. There was a general simulation trend of decreasing efficiency of solar cells versus temperature (S1 Table). It was concluded that moderate temperature rise in the devices with high energy gap of their active layers can help improving the efficiency, while high temperatures are acted upon deteriorating the efficiency and decreasing the devices performance

## Supporting information

S1 Table Data analysis and calculation of the main findings. (XLSX)
